# Recent advances in dental implants

**DOI:** 10.1186/s40902-017-0132-2

**Published:** 2017-11-05

**Authors:** Do Gia Khang Hong, Ji-hyeon Oh

**Affiliations:** Department of Oral and MaxilloFacial Surgery, Dental Hospital, Gangneung-Wonju National University, Gangneung-si, Gangwon-do Korea, Republic of

**Keywords:** Dental implants, Osseointegration, Immediate dental implant loading, Sinus floor augmentation, Computer-aided design

## Abstract

Dental implants are a common treatment for the loss of teeth. This paper summarizes current knowledge on implant surfaces, immediate loading versus conventional loading, short implants, sinus lifting, and custom implants using three-dimensional printing. Most of the implant surface modifications showed good osseointegration results. Regarding biomolecular coatings, which have been recently developed and studied, good results were observed in animal experiments. Immediate loading had similar clinical outcomes compared to conventional loading and can be used as a successful treatment because it has the advantage of reducing treatment times and providing early function and aesthetics. Short implants showed similar clinical outcomes compared to standard implants. A variety of sinus augmentation techniques, grafting materials, and alternative techniques, such as tilted implants, zygomatic implants, and short implants, can be used. With the development of new technologies in three-dimension and computer-aided design/computer-aided manufacturing (CAD/CAM) customized implants can be used as an alternative to conventional implant designs. However, there are limitations due to the lack of long-term studies or clinical studies. A long-term clinical trial and a more predictive study are needed.

## Background

The most common cause of teeth loss is periodontitis, and other causes include dental caries, trauma, developmental defects, and genetic disorders [1]. The use of dental implants to rehabilitate the loss of teeth has increased in the last 30 years [2]. Before dental implants, dentures and bridges were used, but dental implants have become a very popular solution due to the high success rate and predictability of the procedure, as well as its relatively few complications [1, 3].

Many studies related to dental implants have been published and some are in progress. In this paper, current knowledge of dental implants is summarized in each section (implant surface, immediate loading versus conventional loading, short implant, sinus lifting, and custom implant using three-dimensional printing).

## Review

### Implant surface

Modification of the implant surface has been studied and applied to improve biological surface properties favoring osseointegration [4]. The surface roughness of implants has been increased by various methods such as machining, plasma spray coating, grit blasting, acid etching, sandblasted and acid etching (SLA), anodizing, and biomimetic coating [3–6]. The key factor in implant osseointegration is surface roughness, which shows increased osteoblast activity at 1 to 100 μm of the surface roughness compared to a smooth surface [6]. It is believed that rough surfaces have better osseointegration than smooth surfaces, but the results of the research have been diverse and it is not clear that multiple treatments provide better predictive results [7].

The machined implant surface is the first-generation implant surface design with a turned surface implant [4, 7]. Plasma spray coating generally forms a thick layer of deposition such as hydroxyapatite (HA) and titanium by spraying a material dissolved in heat on the surface of the implant [5]. Grit-blasting is a process of spraying particles onto the surface of the implant using ceramic material or silica. Sand, HA, alumina or titanium dioxide (TiO_2_) particles are used and acid etching is performed to remove the remaining blasting particles [5]. Acid-etching is the roughening of the titanium implant surfaces using strong acids such as hydrofluoric acid (HF), nitric acid (HNO_3_), and sulfuric acid (H_2_SO_4_) or combinations of these acids [5]. SLA is acid etching after sandblasting with 250–500 μm large grit particles [7]. Anodizing is the dielectric breakdown of the TiO_2_ layer by applying an increased voltage to generate a micro-arc. This process forms a porous layer on the titanium surface [8].

In the short-term, the survival rate of SLA, HA coating, and oxidized surface modifications was reported to be 100%, but the survival rate tended to be slightly lower in the long-term [9–11]. The long-term survival rate of each surface modification is shown in Table 1. In SLA, the survival rate at 10 years follow-up was 98.8 ~ 99.7% [12, 13] and in titanium plasma sprayed (TPS), the survival rate at 20 years follow-up was 89.5% [14]. With anodizing, the survival rate at 8 ~ 12 years follow-up was 96.5 ~ 100% [15–17]. With HA coating, although the survival rate at 10 years follow-up in 2007 was as low as 82.0% [18], there was also report of 98.5 and 93.2% in published papers in 2000, respectively, which was similar to uncoated titanium implants [19].

**Table 1:** The survival rates by modifications of the implant surface

Author/year	Modification material of implant surface	Follow up	Survival rate
Buser D, et al./2012 [12]	Sandblasted and acid-etched (SLA)	10 years	98.8%
van Velzen FJ, et al./2015 [13]	Sandblasted and acid-etched (SLA)	10 years	99.7%
Chappuis V, et al./2013 [14]	Titanium plasma sprayed (TPS)	20 years	89.5%
Degidi M, et al./2012 [15]	Anodized	10 years	96.5%
Mozzati M, et al./2015 [16]	Oxidized	9–12 years	97.1%
Pozzi A, et al./2014 [17]	Oxidized	8–10 years	100%
Binahmed A, et al./2007 [18]	Hydroxyapatite (HA)	10 years	82.0%
Lee JJ, et al./2000 [19]	Hydroxyapatite (HA)	4–8 years	93.2–98.5%

There are various surface modifications as mentioned above. It is said that any surface modification provides a good surface for osseointegration when the surface roughness is 0.44 ~ 8.68 μm [5]. It is said that acid etching and coating are the most preferred for making good roughness of the implant surface [7]. There is a study that suggested HA is superior to sandblasting, SLA, TPS, and/or machined surfaces in bone-implant contact ratio [20]. On the other hand, there is a study that suggested a bone-to-implant contact of a blasted-etched and covered with HA group was better than a blasted group, acid-etched group, and blasted and acid-etched group; however, there were no significant differences [21].

Recently, research on implant surface modifications using inorganic materials (HA, calcium phosphate, bisphosphonate, etc.), growth factors (bone morphogenetic protein, platelet-derived growth factor, transforming growth factor beta, fibroblast growth factor, vascular endothelial growth factor, etc.), peptides, and extracellular matrix components (collagen, chondroitin sulfate, vitronectin, hyaluronic acid, etc.) has been underway as part of bioactive surface modification [2, 4, 22].

In animal studies, modifications of the implant surface by biomolecular coating seemed to enhance osseointegration by promoting peri-implant bone formation in the early stages of healing, and it seemed to improve histomorphometric analysis and biomechanical testing results [4]. In animal studies, biological coating did not have a statistically significant effect on peri-implant bone growth, but statistically significant effects were observed with inorganic and extracellular matrix component coatings [2]. Furthermore, such modifications of the implant surface do not always provide beneficial effects on osseointegration [4]. Long-term clinical studies are needed.

### Immediate loading versus conventional (delayed) loading

According to many previous studies, many researchers believed that after implantation in the jaw for a future prosthesis, titanium implants should be left submerged to undergo a healing process before they are capable of functional loading. This healing process, which is called osseointegration, could be completely achieved in a period from 3 to 6 months [23]. The reason for the delayed loading was to avoid micro-movement on the implant, which could interfere with the healing process. If this situation occurs, connective tissue can develop at the interface between the implant surface and the bone. The result would be failure of the implant due to not being able to resist the masticatory forces [24].

Following the progressive development of technologies and the wide spread of implantation in dentistry, more recent research has focused on the mechanism of bone healing. It has provided a better understanding of osseointegration [25]. It was suggested that it would be possible to reduce the period between implantation and the placement of a prosthesis [26].

Over the past 20 years, a number of studies and trials have reported similar results with trans-mucosal implants compared with submerged implants. As a result, it is not necessary to submerge the implants under the mucosa during the healing period, which eventually introduced the immediate loading protocol [27, 28].

This protocol was initially developed for the treatment of edentulous patients, and its main purpose was to restore immediate function and aesthetics, which are usually the main concerns of patients [29]. Numerous recent studies that focused on this concept have shown excellent results because the primary outcome was survival of the implant. A study showed an implant survival rate of 91.7% for immediately loaded implants at the 2 years of follow-up [30]. A 100% survival rate was reported in 11 edentulous patients treated with immediate full-arch implants [31].

In studies that compared the immediately loaded implants with conventionally loaded implants, the results showed high survival rates in both groups. The first part of a study about late inter-antral implantation in the nonaugmented edentulous maxilla reported survival rates of 98.3% in the immediate loaded implants group and 96.7% in the conventional group at a mean observation period of 4.7 years [32]. The results in the second part of the study, in cases of immediate inter-antral implantation, also showed similar findings. They were 97.6 and 96.6% for a mean observation period of 3.9 years [33]. A systematic review reported a survival rate of 98.2% in the immediate loading versus 99.6% in the conventional loading when reviewing 29 randomized-control studies [34].

However, when considering the rate of failure between immediate loading and conventional loading in edentulous patients, there were publications that showed a higher risk of failure in treatment with an immediate loading protocol. Another article of meta-analysis showed that immediate loading indicated a slightly higher implant failure rate than conventional loading [35]. A similar finding was also reported, but with a more significant difference [34].

Marginal bone loss (MBL) is also considering as a primary outcome when comparing immediate loading and conventional loading. Progressive MBL was demonstrated as one of the measurements for evaluation of implant failure [36]. There were many recent publications that focused on the comparison of MBL in both implantation of single-tooth cases and edentulous cases.

A minimal MBL with no mobility and peri-implant radiolucency in both treatment modalities were reported when evaluated clinically and radiographically in 20 patients with the need for fixed implant-supported prosthesis for missing mandibular first molars over a period of 72 months [37]. Another study on implantation for single-tooth cases also showed similar findings. There were no significant differences in bone loss between the immediate implant loading and conventional implant loading groups at 1 year follow-up after implantation of a single tooth in the anterior maxilla [38].

This trend could also be found in many studies that focused on edentulous cases. When immediate loading four implants with a pre-existing denture converted to a fixed dental prosthesis compared with conventional loading (3–6 months), it was reported that the same change of 1.2 mm in marginal bone over 5 years in both groups was observed [39]. Also an insignificant difference in mean MBL between the two treatment modalities in both late and immediate inter-antral implantation in the nonaugmented edentulous maxilla was reported [32].

Patient-related outcomes were frequently chosen as a secondary outcome in many publications related to immediate loading versus conventional loading. In a previously mentioned systematic review, most patients preferred immediate loading rather than the conventional loading depending on general and aesthetic satisfaction as well as on postoperative outcomes, such as pain, edema or the need for medications [34]. Other different findings were found. Patients in the immediate loading group reported higher satisfaction than the conventional loading group. However, at the end of a 1 year observation period, functional differences between the two groups had disappeared. Postoperative pain was the only significant difference, with a lower value in the immediate loading groups after the third day [40]. A study, however, showed that immediate loading evoked more postoperative pain on the first day and more swelling on the third day rather compared to the delayed loading. The study compared immediate and delayed loading of single implants to support mandibular overdentures, thus suggesting that the number of implants could affect the decision about whether immediate loading or conventional loading should be considered [41].

Based on the current evidence pool, it could be suggested that immediate loading can be used as a successful treatment modality. It reduces treatment times, provides early function and aesthetics, preserves the alveolar bone as well as prevents unwanted migration of an adjacent tooth in the case of missing a single tooth. However, to achieve the desired treatment outcome, some factors must be taken into consideration when immediate loading is chosen as a treatment procedure (adequate primary stability, patient compliance, and the number of implants).

### Short implant

In an atrophic alveolar ridge, there are many anatomical limitations (maxillary sinus, nasal floor, nasopalatine canal, inferior alveolar canal) that make placement of a standard implant difficult [42]. To overcome these limitations and vertical bone deficits, additional surgical procedures, such as guided bone regeneration, block bone grafting, maxillary sinus lift, distraction osteogenesis, and nerve repositioning, are performed to place a standard implant [42, 43]. However, the procedure is sensitive, challenging, costly, and time-consuming and increases surgical morbidity and causes many complications such as sinusitis, infection, hemorrhage, nerve injury, and gait disturbance [42, 44, 45].

Short implants are considered to be simpler and more effective by reducing the likelihood of such complications, patient discomfort, procedure costs, and procedure times in rehabilitation of the atrophic alveolar ridge [42, 46–49]. The term of a short dental implant is subjective, and there is no clear criteria for the length of a short dental implant [43, 46, 47]. Some articles defined 10 mm or less as the criterion of a short dental implant [47, 50], and some defined less than 10 mm as a short dental implant [46, 51]. Some defined the short implant as 8 mm or less [43, 52, 53]. Implant companies have recently offered short implants of less than 8 mm [47]. In this paper, a short dental implant was defined as less than 8 mm, which is similar to other papers [48, 54–56].

The list of the papers reviewed and the results are shown in Table 2. The papers were published within the last 5 years (from 2013 to 2017) and included dental implants that were less than 8 mm. The period of follow-up ranged from 1 to 5 years. The length of the dental implant varied from 4 to 6.6 mm, and a comparison with long or standard dental implants also varied with and without bone grafts. In this paper, failure was defined as implant loss.

**Table 2:** The survival rate of standard and short implants

Author/year	Length standard implants and number of implants	Length short implants and number of implants	Diameter (Ø mm)	Follow up	Survival rate standard implants	Survival rate short implants
Pohl V, et al./2017 [54]	11, 13, 15 mm 68	6 mm 61	4 mm	3 years	100%	100%
Rossi F, et al./2016 [57]	10 mm 30	6 mm 30	4.1 mm	5 years	96.7%	86.7%
Felice P, et al./2016 [58]	≥ 8.5 mm 116	4 mm 124	4 mm	1 year	98.28%	97.58%
Romeo E, et al./2014 [59]	10 mm 19	6 mm 21	4 mm	5 years	100%	95.24%
Pistilli R, et al./2013 [60]	≥ 10 mm 91	6 mm 80	4 mm	1 year	96.7%	100%
Pistilli R, et al./2013 [61]	≥ 10 mm 69	5 mm 68	5 mm	1 year	98.55%	97.1%
Gulje F, et al./2013 [62]	11 mm 101	6 mm 107	4 mm	1 year	99.01%	97.2%
Esposito M, et al./2014 [63]	≥ 10 mm 68	5 mm 60	Standard : 4 and 6 mm Short : 6 mm	3 years	97.06%	91.67%
Felice P, et al./2014 [64]	≥ 9.6 mm 61	6.6 mm 60	4 mm	5 years	95.08%	91.67%

The clinical outcome of short implants in these various criteria is controversial. The lower survival rate of 86.7% for 6-mm short implants after 5 years was reported [57]. On the other hand, the survival rate of 100% for 6-mm short implants after 3 years [54] and the survival rate of 97.6% for 4-mm short implants after 1 year were reported [58]. The survival rate of 95.2% for 6-mm short implants after 5 years [59], and the survival rate of 100% for 6-mm short implants after 1 year were reported [60]. The survival rate of 97.1% for 5-mm short implants after 1 year [61], and the survival rate of 97.2% for 6-mm short implants after 1 year were reported [62].

In studies comparing standard implants without a bone graft and short implants, the survival rate ranged from 86.7 to 97.6% [57–59]. In studies comparing standard implants with a bone graft and short implants, the survival rate ranged from 91.7 to 100% [54, 60, 61, 63, 64]. There was also a statistically significant higher incidence of complications in the group with a standard implant with a bone graft [60, 64]. In addition, there was also statistically significant higher marginal bone loss in the group with a standard implant with a bone graft [63, 64].

Recent studies have indicated that single-crown implants in the posterior region can be considered as a predictable treatment option [51, 65, 66]. However, the implant placement on type IV bone or with the length of 8 mm or less should be used with caution, because of the higher risk of failure compared to the standard implant [65, 66].

In conclusion, the use of a short implant of less than 8 mm had similar clinical outcomes compared with a standard implant, but long-term follow-up data for more than 5 years is needed.

### Sinus lifting

#### Sinus augmentation technique

Sinus augmentation, in other words, sinus lifting was first described as a surgical technique for creating a bone window in the vestibular wall of the sinus. After that, the sinus epithelium was gently raised to create a space for bone grafting. Bone harvesting was performed in the iliac crest area and then placed in the prepared space. The healing period took about 6 months before implantation [67]. The use of autogenous bone, allograft and alloplast material for bone grafting during sinus augmentation was suggested. In addition, the one-stage approach was demonstrated, in which sinus augmentation and implantation are performed in one surgery while the two-stage approach had the implantation taking place after several months of sinus augmentation [68]. The abovementioned technique has been known as sinus lifting with the lateral window and is still widely used in modern implant dentistry due to its reliable efficiency.

Osteotome sinus floor elevation was a less invasive one-stage technique. In this technique, the sinus epithelium was accessed via a crestal approach. The tip of the osteotomes, with increasing diameter, push a mass of bone to a required level that beyond the original sinus floor, eventually elevating the sinus epithelium. The implants were then inserted without drilling after sinus augmentation, followed by bone grafting if necessary. However, it was suggested that a minimum of 6-mm alveolar bone height was needed for primary stability [69].

One of the most common complications of sinus augmentation was perforation of the sinus epithelium, which could be a result of sinusitis, excessive bleeding and delayed healing. Many modified techniques and surgical instruments were introduced to avoid complications of sinus augmentation. A crestal approach using a non-traumatic drill to decrease the risk of tearing the sinus membrane was suggested. In retrospective study, long implants (13 mm and 15 mm) were inserted in 265 cases. For bone grafting, many options were available. The bone can be harvested from the osteotomy site, or a bone substitute can be used. In the case of experienced surgeons, implants could be inserted without grafting, and the tip of the implants could act as a support for the sinus membrane. Similar to crestal approach technique, a primary stability is achieved if a minimum of 3 mm of alveolar bone height is available [70].

In addition, there were good results with the use of absorbable collagen membranes in perforated sinus for sinus elevation and implant placement [71]. In other technique for treatment of the posterior edentulous maxilla, implants were first placed in the ulna. After 6 weeks, bone blocks containing implants were harvested and transplanted into the sinus area protruding 3 to 4 mm. Implants were then left to heal for 6 weeks. To compare the efficiency of this treatment modality, patients treated with particulate bone grafts (an autogenous bone graft from the symphysis, tibia, or iliac crest) acted as controls. Grafts were allowed to heal for 6 months before implantation in the control group. There were no differences between the two groups in terms of implant stability. There was a significant increase in implant stability at 6 and 12 months in both groups. An ulna implant block, in combination with sinus grafting, could be an effective solution for increasing the vertical bone height, especially in severe cases of bone atrophy [72].

#### Grafting materials

In terms of grafting materials, the autogenous graft is considered to be the most predictable and reliable source of grafting for the replacement of deficient bones. The characteristics of the autogenous bone graft are that they are osteoconductive, osteoinductive, and osteogenic, and hardly any other grafting materials from other sources have the same capabilities. Intra-oral donor sites are convenient to harvest and share the same biological and molecular structures with the recipient site but yield a limited volume. Extra-oral donor sites could provide a significant volume of grafting material, but there is an increase in surgical complexity, morbidity, and scarring [73]. Therefore, bone substitutes have been developed to further increase the option for choosing grafting materials.

Allografts consist of ‘same species’ tissue, which is harvested from cadaveric bone and undergoes various procedures to reduce antigenicity. Xenografts consist of different species tissue. The organic components are removed to create a mineral scaffold containing residual collagen. Alloplasts are synthetic bone substitutes. There are many types, which are classified by porosity. These graft materials could be manufactured as bone particles or large blocks can be mixed with autogenous bone [45].

Following the introduction of many types of grafting material, a controversy arose focusing on the question of which material should be chosen as the best solution for grafting augmentation and the related procedures.

The osseointegration of micro-implants was compared when performing sinus augmentation with the use of one of the three types of grafting materials: autogenous bone, bovine hydroxyapatite (BH), or mixture of BH with autogenous bone. The results of clinical and histological evaluations concluded that there were no statistically significant differences between any of the grafting materials; however, it was suggested that adding autogenous bone might accelerate the healing time [74]. This similar trend was also demonstrated. A randomized controlled trial (RCT) was conducted to compare the effectiveness between pure bovine bone matrix grafts with pure autogenous grafts. The final results also suggested that using bovine bone matrix grafts or autogenous grafts yielded no differences in terms of the implant or prosthetic failure, complications, discomfort, and bone level; however, there was an increase in operating times for autogenous bone grafts. The reason could be it required a longer time for the bone harvesting procedure [75].

With the advancement in genetic and molecular research, numerous studies have been conducted in the past decade to establish a better understanding of the efficiency, safety, and mechanism characteristics of recombinant human bone morphogenetic protein-2 (rhBMP-2), which is an osteoinductive protein that is essential for bone growth and regeneration. Some of the growth factors, platelet-rich plasma (PRP) and other molecules, were found [76]. Many types of research were conducted to determine the effectiveness of using grafting material with the addition of rhBMP-2 in sinus augmentation for enhancing osseointegration.

A study aimed to determine whether the use of PRP could have a positive effect on osseointegration of autogenous bone grafts used for sinus augmentation. Both maxillary sinuses in five edentulous patients were augmented with an autogenous bone graft. PRP was only added to one grafting site. Micro-radiographical and histomorphological examination revealed no significant difference between the PRP and non-PRP sides, suggesting that PRP has no useful characteristic in promoting healing of autogenous bone grafting [77]. In animal study, the bovine bone graft with PRP had less new bone formation and bone healing process than xenograft alone [78]. On the other hand, there was a study to confirm the effect of using PRP when using the bovine bone as the grafting material in a RCT. Patients underwent sinus augmentation with bovine bone graft alone or bovine bone graft with PRP. Additionally, a split-mouth study was conducted, which performed histological evaluation. Analysis of the results revealed that grafting sites treated with PRP showed better bone remodeling, suggesting the possibility of an increase in the new volume of bone when PRP is used with bovine bone grafting [79].

#### Alternative techniques

Despite the reliability and efficiency of various sinus augmentation techniques, there is still a high rate of complications and complexity for such procedures. With the advances in technology and improvements in design and manufacture of implants, some alternative concepts suggested implantation without sinus augmentation could be possible.

The use of a tilted (angulated) implant in the posterior maxilla was suggested to avoid sinus augmentation. In this study, an evaluation was made to compare the efficiency between tilted and axial implants with no sinus grafting. After 5 years of follow-up, the implant success rate was 95.2% (survival: rate 100%) for the tilted implants and 91.3% (survival rate 96.5%) for the axial implants. The average marginal bone loss was 1.21 mm for the tilted implants and 0.92 mm for the axial ones [80].

**Fig. 1:**
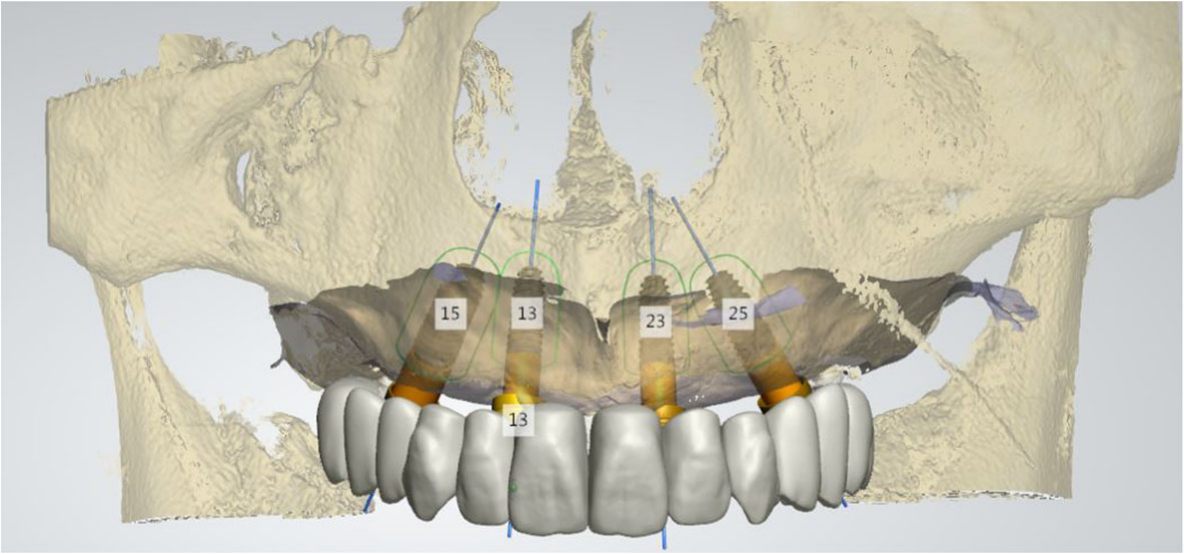
The all-on-4 concept for complete edentulism. It is a concept that rehabilitates the complete edentulism using four implants. The anterior implants are placed vertically and the posterior implants are tilted to avoid anatomical structures such as the maxillary sinus

The concept of using tilt implant was further enhanced. Trans-sinus tilted implants, with the implant body inside the sinus, were utilized in the All-on-4 concept for complete edentulous maxilla patients (Fig. 1). A survival rate of 96.4% was achieved at the implant level. The survival rate of prostheses was 100%. Sinusitis occurred in two patients (2.9%). The high survival rate and low complication rate suggest that trans-sinus implants could be an alternative solution to avoid sinus augmentation [81].

Zygomatic implants offer another option treatment modality to sinus augmentation. Almost similar to trans-sinus tilted implants, zygomatic implants are long implants that pass through the sinus or laterally to the sinus [82]. The difference was the anchorage position. While the tip of a trans-sinus tilted implant is positioned in the bone between the anterior sinus wall and the nasal cortical bone [81], a zygomatic implant will anchor itself into the zygomatic process for stability.

The use of a short implant (4 to 8 mm long) was also an interesting and straight forward alternate treatment modality for sinus augmentation followed by longer implant placement. In a recent systematic review, there was further clarification of this concept. Eight RCTs from an initial search count of 851 titles were selected, and data extraction was performed. Both long-term follow-up (16–18 months) and short-term follow-up (8–9 months) study showed no significant differences when comparing implant survival rates. Most common complications were membrane perforations, and they were almost three times higher for longer implants in the augmented sinus compared to shorter implants. Morbidity, surgical time, and cost-effectiveness also showed more favorable data in the shorter implant group [83].

Sinus augmentation is the most common indication associated with implant placement in patients with severe edentulous maxilla. With the advancement of implant dentistry, there have been introductions of new techniques and grafting materials, which were aimed to improve the treatment outcomes of sinus augmentation. Several new concepts, such as the use of an angulated implant, zygomatic implant, or short implant, could provide another option for implantation in the posterior maxilla without the need for sinus augmentation, thus making treatment time shorter and reducing the rate of complications and the complexity of the treatment procedure.

### Custom implant using three-dimensional printing

Custom implant using three-dimensional printing (3DP) was first used in the fields of rapid tooling and rapid prototyping. Initially, specifically single, personalized objects were manufactured by 3DP in restorative dentistry. By combining oral scanning with a CAD/CAM design and using 3DP, dental labs can produce dental prostheses (crowns, bridges) and plaster/stone models more rapidly and with excellent precision than most tradition procedures performed by lab technicians [84].

With the advancement of implant dentistry, there was an increase in utilizing CAD/CAM as a supportive means to maximize the results of implant treatment. Customized implant abutments have been successfully produced using CAD/CAM for difficult cases when standard abutments may not provide a suitable option for a future prosthesis. Thus, to combine with customized abutments, customized coping was also manufactured for such cases to provide a more accurate impression [85].

**Fig. 2:**
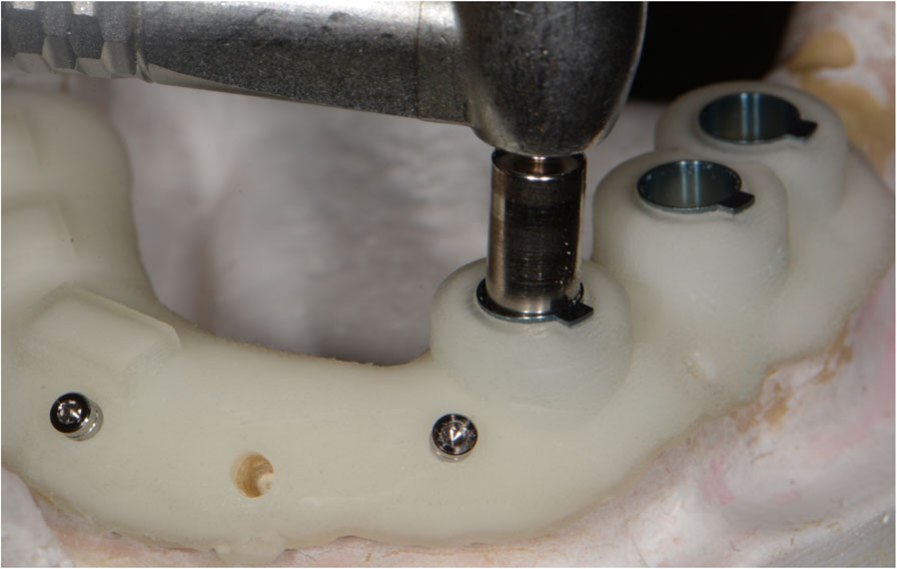
The surgical guide for implant placement. CBCT and CAD/CAM are used to produce a surgical guide for implant placement

In addition to the usage of 3DP and CAD/CAM in the making of prosthesis-related components, some have presented concepts of utilizing this advanced technology in the planning phase of implantation. The use of cone beam computed tomography (CBCT) combined with CAD/CAM was suggested to produce a surgical guide for implant placement (Fig. 2). In this scenario, mini-implants were used as reference points. A software created the three-dimensional simulation and allowed the clinician to plan an ideal implant placement, virtually integrating the future prosthetic for a complete rehabilitation treatment. A digital file of the surgical template was exported, and fabrication of surgical guide was performed by 3DP [86]. There was a study showing favorable results in the accuracy evaluation of computer-guided implant surgery [87].

3DP and CAD/CAM has involved itself in almost every aspect of implant dentistry, from the planning phase to finalizing the prosthesis. The only component left is the implant itself, which is still commonly manufactured by traditional methods. One of the new possible theories with 3DP technology is to produce a customized implant with the analog that mimics the root of the missing tooth, as an alternative to the traditional implant design (threaded, straight, or tapered). With similar dimensions to the original root, the customized implant could provide better matching with the root socket [88]. Recently, many types of research have further explained that this theory have been conducted on cadaver models, animal models, or in clinical trials.

There was an experiment to clinically and histologically evaluate the customized implant placed in an already extracted socket in monkeys. After the extraction of the single-root teeth (upper central and lateral incisors), fabrication of the customized implant was performed with a CAD/CAM system after the root was machine copied to a titanium analog. The implants were then inserted into the respective sockets. Histological findings showed an average mineralized bone-to-implant contact of 41.2 ± 20.6%, suggesting that osseointegration could occur after the placement of titanium implants created by a laser-copy machine [89].

With a more sophisticated study design, the effectiveness of customized zirconia implants with two different surface modifications was compared in 18 patients. The customized implants were fabricated after the extraction of the corresponding teeth. The implant surface then underwent the sandblasting process. However, in group 1 (*n* = 12), implants were modified with additional macro retention while the implants for the other group (*n* = 6) were not. No complications occurred during the healing period. All implants without additional macro retention were lost within 2 months. In the other group, the overall survival rate was 92%. As a result, it could be confirmed that customized zirconia implants, with specific modifications, could achieve primary stability and osseointegration [90].

However, it should be noted that two of the previous studies used a concept of fabricating the customized implant based on the three-dimensional (3D) data of an already extracted tooth. Thus, it could be indicated that, in the cases of a patient requiring implant replacement for a single-tooth, the tooth has to be extracted as the first surgery, and only then could implantation be performed later in another surgery. It would seem more efficient to have the customized implant ready before tooth extraction, allowing immediate implantation and omitting the need for a second surgery. A question arose about whether a pre-extracted tooth or a post-extracted tooth could provide more accurate 3D data as a basic model for the fabrication of a customized implant.

There was a study that compared the accuracy of a customized implant created by 3DP and a fused deposition modeling technique (FDM) based on the pre-extraction CBCT data of the tooth (in vivo) with the real original tooth after extraction (in vitro) from orthodontic patients. The 3D deviations between the in vivo teeth, in vitro teeth, and the 3DP customized implant were compared using studio software. According to the results, an independent *t* test showed that no statistically significant difference was observed between the in vitro teeth and in vivo teeth in terms of average deviation. It could be concluded that with the combination of 3DP and FDM, CBCT data of a pre-extracted tooth could be used for fabricating the corresponding customized implants with high precision as an alternative to 3D data of the post-extraction tooth [91].

A study with a similar design and method was also conducted, with data collected from a human cadaver. After comparisons, the results showed that the greatest differences between the customized implant and the optical scan of the extracted tooth were observed at the apex and the cement-enamel junction (CEJ) areas on the buccal and lingual side. There was an overall decrease in the surface area of 6.33% for the customized implant compared to the original tooth [88].

In addition to the decision of choosing the optimal 3D data between the pre-extraction or post-extraction tooth for fabrication of a customized implant, the intactness of the tooth must also be taken into account, particularly in the root area. Teeth that need to be replaced by implants are commonly damaged or even already extracted; thus, it is suggested that recreating a 3D model based on the contra-lateral tooth could be a suitable option. Additionally, the concept of using 3D data of the tooth without extraction could achieve better accuracy because there was no damage to the tooth by the elevator or dental forceps [88].

With the ongoing development of new technology in 3D and CAD/CAM, it is predicted that customized implants could be the promising future of implant dentistry as an alternative to conventional implant designs. However, more clinical trials are needed to evaluate the effectiveness of this approach.

## Conclusion

Recent findings about surface modifications, immediate loading, short implants, sinus lifting, and custom implants have improved the success rate of implants regarding. However, there are limitations due to the lack of long-term or clinical studies. A long-term clinical trial and a more predictive study are needed.
